# Metagenomics Reveals That Intravenous Injection of Beta-Hydroxybutyric Acid (BHBA) Disturbs the Nasopharynx Microflora and Increases the Risk of Respiratory Diseases

**DOI:** 10.3389/fmicb.2020.630280

**Published:** 2021-02-05

**Authors:** Jiancheng Qi, Dongjie Cai, Yaocheng Cui, Tianyu Tan, Huawei Zou, Wei Guo, Yue Xie, Hongrui Guo, Shi-Yi Chen, Xiaoping Ma, Liping Gou, Hengmin Cui, Yi Geng, Ming Zhang, Gang Ye, Zhijun Zhong, Zhihua Ren, Yanchun Hu, Ya Wang, Junliang Deng, Shumin YU, Suizhong Cao, Metha Wanapat, Jing Fang, Zhisheng Wang, Zhicai Zuo

**Affiliations:** ^1^College of Veterinary Medicine, Sichuan Agricultural University, Chengdu, China; ^2^Institute of Animal Nutrition, Sichuan Agricultural University, Chengdu, China; ^3^Department of Clinical Laboratory, Chengdu Medical College, Chengdu, China; ^4^College of Animal Science and Technology, Sichuan Agricultural University, Chengdu, China; ^5^Tropical Feed Resources Research and Development Center (TROFREC), Department of Animal Science, Faculty of Agriculture, Khon Kaen University, Khon Kaen, Thailand

**Keywords:** starvation, nasopharynx microbiota, respiratory tract, microbial diversity, metagenomics, yak

## Abstract

It is widely accepted that maintenance of microbial diversity is essential for the health of the respiratory tract; however, there are limited reports on the correlation between starvation and respiratory tract microbial diversity. In the present study, saline/β-hydroxybutyric acid (BHBA) intravenous injection after dietary restriction was used to imitate different degrees of starvation. A total of 13 healthy male yaks were imposed to different dietary restrictions and intravenous injections, and their nasopharyngeal microbiota profiles were obtained by metagenomic shotgun sequencing. In healthy yaks, the main dominant phyla were *Proteobacteria* (33.0%), *Firmicutes* (22.6%), *Bacteroidetes* (17.2%), and *Actinobacteria* (13.2%); the most dominated species was *Clostridium botulinum* (10.8%). It was found that 9 days of dietary restriction and 2 days of BHBA injection (imitating severe starvation) significantly decreased the microbial diversity and disturbed its structure and functional composition, which increased the risk of respiratory diseases. This study also implied that oral bacteria played an important role in maintaining nasopharynx microbial homeostasis. In this study, the correlation between starvation and nasopharynx microbial diversity and its potential mechanism was investigated for the first time, providing new ideas for the prevention of respiratory diseases.

## Introduction

Yak (*Bos grunniens*) is semidomesticated herbivore livestock on the Qinghai-Tibet Plateau (Qiu et al., 2012). In the cold seasons, yaks often suffer from severe starvation, weight loss, high morbidity, and mortality due to long-time lack of pasturage (Xue et al., 2005). Recently, it is widely accepted that this high prevalence of diseases was associated with the harsh environment in the cold seasons (Xue et al., 2005). However, it is unclear whether starvation affects the respiratory system and then results in the high prevalence of diseases.

The respiratory tract is an important part of the respiratory system, which harbors various microbial communities in every ecological niche (Dickson et al., 2016). The respiratory tract microbiome is highly dynamic and affected by many factors. For instance, Woldehiwet et al. (1990) found that the upper respiratory tract microbiota of calves was affected by individual differences, age, and environmental temperature; Anand and Mande (2018) summarized that diet could affect respiratory microbiota, whereas Bosch et al. (2016) indicated that upper respiratory tract microbiota in infancy was affected by delivery mode. It was thought that these factors affected the proliferation ability of certain bacteria and immunity of the host (Man et al., 2017). In recent years, the correlations between microbiota and the healthy of respiratory tract has attracted lots of attention. Man and Wypych both believed that airway-related diseases of both humans and cattle are caused by the disturbance of the microbiome (Man et al., 2017; Wypych et al., 2019), and Mohamed summarized that the mucosal microbiota had substantial effects on the bovine respiratory health (Zeineldin et al., 2019). Collectively, it is recognized that maintaining the respiratory tract microbiota homeostasis played a vital role in keeping the airway healthy (Man et al., 2017). There are also many reports on the microbiota differences along the respiratory tract. Bassis et al. (2015) and Nicola et al. (2017) found that the microbiome of the lower respiratory tract was closely associated with that of the upper respiratory tract. Charlson et al. (2011) and Zeineldin et al. (2017) found that the microbiota in the nasopharynx could affect the health of the entire respiratory tract. The nasopharynx is the overlapping area of the oral cavity, nasal cavity, and trachea, which explains why the nasopharyngeal microbiota has a considerable overlap microbial composition of anterior nares, nasopharynx, oropharynx, and trachea, including *Moraxella*, *Dolosigranulum*, *Staphylococcus*, *Corynebacterium*, etc. (Dickson et al., 2017; Man et al., 2017). Therefore, the microbial community profiles of the nasopharynx can reflect the comprehensive situation of the entire respiratory tract (McMullen et al., 2020).

Traditional bacterial culture technology, 16S RNA sequencing, etc., were once used to study nasopharyngeal microbial communities, but these tools have their disadvantages; for example, many bacteria are uncultivable, and 16S RNA sequencing is not deep enough to be accurate to species level. In the past decades, with the advances in the next-generation sequencing technologies, metagenomics-based studies have been widely applied to determine the composition of various microbiomes and to analyze their functions at the DNA and RNA levels (Wang et al., 2015; Gilbert et al., 2016). Metagenomics can accurately detect all the species and their relative abundance in a sample and allow us to precisely analyze and predict the structure and function of the microbial community.

Previous research showed that the main features of starvation are lower blood glucose and elevated blood ketones [acetone, acetoacetate, and especially β-hydroxybutyric acid (BHBA)] due to the fulsome catabolism of fat (Whiting et al., 2012; Dhatariya et al., 2020). BHBA, the most primary (>70%) end products of lipid decomposition, provides energy for animals when animals suffer excessive starvation (Belkhou et al., 1991; Cahill, 2006; Dhatariya et al., 2020).

Hence, to explore the correlation between starvation and nasopharyngeal microbiota, we imitated mild and excessive starvation state by fasting and BHBA solution intravenous infusion. The nasopharyngeal microbiome was sampled and sequenced, and their differences in diversity, structure, and function among the experimental and control groups were analyzed using a metagenomic shotgun sequencing approach. Until now, there are limited studies on the correlation between starvation and respiratory tract microbiome; our study will fill this knowledge gap, enrich our understanding of the microbiota of the respiratory tract, and provide new prevention and treatment strategies for respiratory diseases.

## Materials and Methods

### Experimental Animals

Before the experiment, 13 healthy (with no macroscopic symptoms) and well-grown (with similar weights, 237.97 ± 11.75 kg) 2.5-year-old male Jiulong yaks were adaptively fed (without any antibacterial agents) for 2 months in independent cowsheds. All cowsheds were cleaned with insect repellant and sanitizer every week. All fodder and water were prepared according to Zou et al.s’ (2020) study. After adaptively feeding, all yaks (*n* = 13) were randomly divided into three groups: control group (*n* = 3), mild dietary restriction (DR) group (*n* = 5), and excessive DR (*n* = 5) group. Yaks in the control group were numbered Z1–Z3, yaks in the mild DR group were numbered G1–G5, and yaks in the excessive DR group were numbered GB1–GB5. Yaks in the mild DR group and excessive DR group were starved (without any fodder) for 9 days. The 9 days of dietary restriction time was determined according to previous work (Zarrin et al., 2013; Zou et al.s’, 2020) to ensure that yaks were in the state of negative energy balance without health threatening. Yaks in the control group were free to access fodder within the synchronous 9 days. All yaks received a continuous 48 hours of intravenous infusion from 9:00 AM on the seventh day to 9:00 AM on the ninth day. Yaks in the control group and mild DR group were infused with 0.9% saline, whereas yaks in the excessive DR group were infused with BHBA solution (1.7 mmol/L). The experiment flow before sampling is visualized in Supplementary Figure 1B.

### BHBA Infusion

The BHBA solution was prepared following the previous study (Zarrin et al., 2013). BHBA acid sodium salt (Sigma, United States) was solvated into ultrapure water to the concentration of 1.7 mmol/L. The pH value of this solution was adjusted to 7.4 by HCl followed by autoclaving at 131°C, 100 kPa for 50 min. Then, the prepared solution was stored at 4°C as soon as being filtered through the 0.22-μm filter. The indwelling intravenous catheters (Jinhuan Medical Supplies, China) were fitted on both of the ear veins of each yak on day 7. The infusion was through the left-side catheters of yaks by a peristaltic pump (Haoke Medical Instrument, China). The initial infusion dose was calculated based on the bodyweight of yaks (8.5 μmol/Kg/min). During the first 2 h of infusion, the blood samples were collected through the right-side catheters every 15 min and then determined the BHBA concentration immediately using a blood ketone meter (Dizhun Biotechnology, China). BHBA infusion rate was instantly adjusted to maintain the blood BHBA concentration between 1.5 and 2.0 mmol/L, whose aim was to avoid ketosis caused by excess high BHBA concentration. The yaks in the control group and mild DR group were infused into 0.9% saline solution with the same infusion time and rate. More details could be found in Zou et al.s’ (2020) work.

### Sample Collection, DNA Extraction, Sequencing, and Quality Control

When the intravenous infusion stopped, microbiota samples were collected using 20-cm sterile deep nasopharyngeal swabs (Merlin Technology, China) from the nasopharynx mucosa and immediately stored in a dry icebox. DNA was extracted using the MO BIO PowerSoil DNA Isolation Kit (MO BIO Laboratories, United States) according to Earth Microbiome Project standard protocols (Marotz et al., 2017). DNA concentrations of all samples was detected by NanoDrop (Thermo Scientific, United States), and the results ranged from 15.2 to 75.4 ng/μL. DNA samples’ quality was estimated on agarose gel electrophoresis. Only samples that meet the following criteria were used for library construction: (1) DNA concentration is >15 ng/μL; (2) the total weight of DNA is >6 μg; (3) DNA band that was visualized on agarose gel electrophoresis must be clear and of good quality. Finally, 1 μg DNA of each sample was pooled to an equimolar concentration to construct the DNA libraries (DNA was sheared to 350 bp) using the Illumina DNA Sample Preparation Kit according to the manufacturer’s instructions. Amplified libraries were sequenced on Illumina HiSeq 2500 platform (2 × 250 bp). Adaptor contamination was removed using Cutadapt 1.3 (Martin, 2011) with parameters “-o 4 -e 0.1.” Quality control was performed using a sliding window (5-bp bases) in Trimmomatic (Bolger et al., 2014) with the following criteria: (1) cutting once the average quality within the window falls below Q 20; (2) clean reads do not contain any N bases; (3) trimming is applied to the 3’ end of reads, dropping those reads that were of less than 50-bp length; (4) only paired-end reads were retained for downstream analyses. To contigs and scaffolds, the obtained paired-end clean reads of each sample were performed *de novo* assembly using Megahit with the parameter “K-mer∼ [27, 127]” (Li et al., 2010). Detailed contigs/scaffolds statistical information was shown in Supplementary Table 8 (Sheet 2).

The metagenome dataset used in this study was deposited into the National Centre for Biotechnology Information’s Sequence Read Archive (SRA; http://www.ncbi.nlm.nih.gov/sra) under accession bioproject number: PRJNA681085 (SRA: SAMN16932244-SAMN16932256)^1^.

### Species Annotation

To analyze the species composition, the scaffolds/scaftigs of each sample were subjected to BLASTN (“E < 0.00001”) against the bacterial, archaeal, fungal, and virus sequences in the NCBI-NT database (National Centre for Biotechnology Information–Nucleotide Collection, v2016-6-19). Because each target sequence could match different reference sequences that belong to a different taxon, we performed the Lowest Common Ancestor algorithm (Huson et al., 2018) using MEGAN5 (MEta Genome Analyzer) (Huson et al., 2011) software to increase the preciseness and dependability without loss of biological significance. In brief, we classified the last level of common classification before the reference sequences branched into different species as the annotation information of species classification of the target sequences. Then the relative abundance of each taxon at every classification level was obtained by combining the relative abundance of these scaffolds/scaftigs sequences in each sample using Quantitative Insights Into Microbial (QIIME) software (Caporaso et al., 2010). To analyze the significance of species relative abundance difference, we performed a two-tailed *t*-test against the average relative abundance using the SciPy database (Virtanen et al., 2020) in Python software and controlled the false discovery rate (FDR) using the Benjamini-Hochberg method (Benjamini and Hochberg, 1995). In brief, we calculated the fold change value of every functional group between each sample and demonstrated them using Log_2_ (fold-change value). Only those functional groups with both | Log2 (fold-change value) | > 1 and *p* < 0.05 were considered having significant difference. According to the composition structure of each sample at each classification level and their relative abundance, we visualized them in heat map using R software package. Through randomly sampling a certain number of sequences in each sample, we predicted the possible species total number and their relative abundance within a set of given sequencing depths and drawn rarefaction curve (Heck et al., 1975) using QIIME software. To analyze the distribution of species abundance, the taxon of each sample at species level was arranged from high to low according to their relative abundance, and the relative abundance value was transformed into vertical ordinate by Log_2_, then we drew the rank abundance curve using R software. We also calculated the Spearman rank correlation coefficient (Sedgwick, 2014) of the top 50 species with the highest relative abundance using Mothur software (Schloss et al., 2009) and drew the connection networks (Faust and Raes, 2012) of species (| ρ| > 0.8, *p* < 0.01) and visualized them using Cytoscape software (Shannon et al., 2003). To compare the diversity of different samples and correct the diversity difference caused by the sequencing depth, we randomly sampled the bottom functional group abundance spectrum of all samples in each functional database or the species-level composition spectrum according to the lowest sequencing depth. And then we obtained the alpha diversity (including Chao1 index, ACE index, Shannon index, and Simpson index) of each sample by QIIME software. To analyze the unsupervised β diversity, principal complement analysis (Euclidean distance) (Ramette, 2007) was performed on the abundance spectrum of the bottom functional groups and species-level composition spectrum annotated by each functional database in each sample using QIIME software and R software. At last, to find a biomarker, we performed linear discriminant analysis effect size (LEfSe) analysis (Segata et al., 2011) by submitting the composition spectrum data at species level to Galaxy online analysis platform (huttenhower.sph.harvard.edu/galaxy/).

### Function Annotation

Scaffolds/scaftigs sequences with more than 200 bp of each sample were selected to predict genes at the MetaGeneMark database (Zhu et al., 2010), and then we identified the open reading frames and obtained the predicted protein sequences. CD-HIT (Cluster Database at High Identity With Tolerance) (Fu et al., 2012) was used to classify the obtained protein sequences based on 90% sequence similarity and to remove redundancy, and the longest sequence was selected as the representative sequence to obtain the non-redundant protein sequence sets. We used Soap.coverage (soap.genomics.org.cn/) to determine the relative abundance of each protein of each sample. By comparing the protein sequence sets with the Kyoto Encyclopedia of Genes and Genomes (KEGG) Pathway Database (Kanehisa et al., 2004), the proteins predicted by the MetaGeneMark database could be annotated and classified. In brief, the non-redundant protein sequence sets were uploaded to KEGG Automatic Annotation Server for functional annotation (in “GENES data set,” partially select “for Prokaryotes”; the rest of the parameters are default), and the returned annotation results were summarized so that the annotation results at each classification level and their corresponding relative abundance were obtained. And then we obtained the relative abundance distribution of each functional classification level at each sample in the KEGG database using QIIME software. The enrichment analysis results were obtained by hypergeometric distribution in the SciPy by a two-tailed *t*-test against KEGG Orthology (KO) functional groups, and the FDR was controlled.

### Statistical Analysis

Relative abundances of the non-eukaryotic KO gene were calculated by normalizing all the KOs of each sample to sum to 1. Observation matrix tables containing relative abundance information of KOs were used to calculate Euclidean distance based on UPGMA algorithm, and principal coordinates analysis plot was built using the R data analysis package. The entire visualized figures were drawn by an R package. The test of significance based on a two-tailed *t*-test was performed to determine whether there was a significant difference in abundance of the gene between different diet groups by using GraphPad Prism 5 software (GraphPad Software, Inc., United States).

## Results

### Data Quality and Diversity Analysis

All DNA samples of 13 nasopharyngeal swab samples were qualified to be added to Illumina HiSeq 2500 high-throughput sequencing platform. Then, the total metagenomic DNA was randomly interrupted into short clips, which were subjected to paired-end sequencing (2 × 250 bp) for library construction. A total of 678,219,000 raw paired-end reads were generated, and the average proportion of these sequences with high-quality reads in each sample was 99.86 ± 0.01. Quality control analysis showed that the assembled sequences were of high accuracy, which made subsequent analysis results reliable enough. The detailed indexes of quality control are shown in Supplementary Table 1A.

**FIGURE 1:**
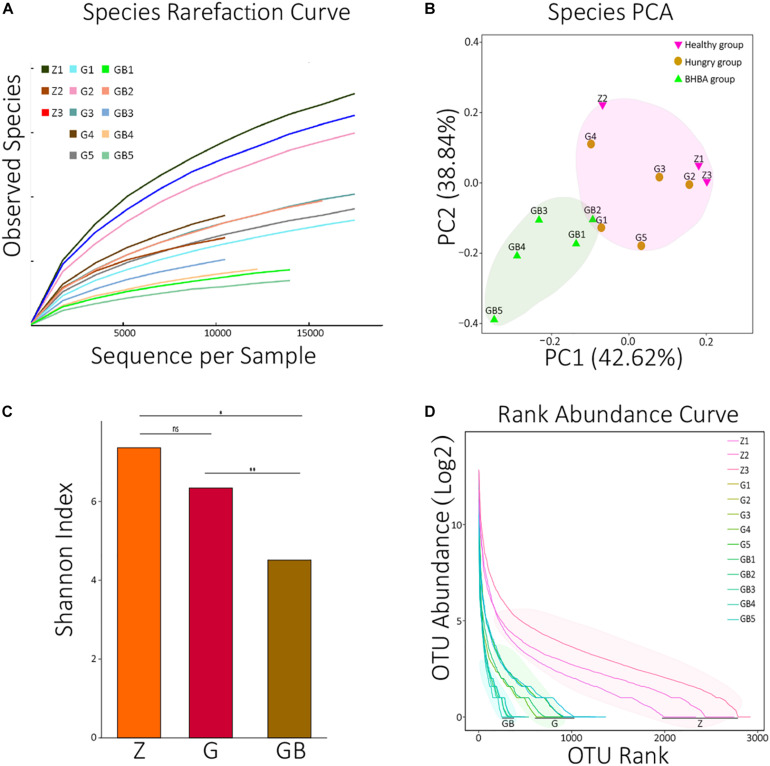
Data processing and diversity analyses. **(A)** Species rarefaction curves of 13 samples. X: number of randomly selected sequences in each sample, Y: species number observed at corresponding depth. The curvature of the curve represents the possibility that observes new species, the smoother curves, the lower probability of observing new species, and the deeper sequencing depth. **(B)** PCA (principal component analysis) of species in each sample. The distance between the two samples represents the significance of the difference between them. **(C)** The average means of Shannon diversity index of each group. ns: non-significant, *p* > 0.05; *0.01 < *p* < 0.05; **p < 0.01. **(D)** The rank abundance curves of each sample at the species level. X: taxon arranged in the order of abundance at the species level, Y: the Log2 means which translated from the relative abundance of each species in the corresponding sample. The length of the curve represents the richness of each sample; the curvature of the curve represents the evenness of each sample. OTUs: operational taxonomic units; two sequences with >97% similarity at the species level were defined as the same OTU.

In the rarefaction curve, before reaching 18,000, the rarefaction curves of 13 samples all fractured and trended to flatten out, indicating that the sequencing depth was sufficient to reveal their microorganism composition (Figure 1A). We performed principal component analysis (PCA, Euclidean distance) against all species of 13 samples. It was found that although there were differences among the 13 samples, they could be roughly clustered into two populations (pink and reseda areas) (Figure 1B). A similar result was also found in the PCA of KO analysis (Supplementary Figure 1A).

Then, we calculated the Shannon diversity index of all 13 samples and visualized the average means of each group using an R package (Supplementary Table 1B and Figure 1C). There was no significant difference between the control group and mild DR group (*p* > 0.05); the Shannon index of the control group was significantly higher than that of the excessive DR group (*p* < 0.05), and the Shannon index of the mild DR group was extremely higher than the excessive DR group (*p* < 0.01). The detailed statistical data of diversity indexes are shown in Supplementary Table 1B. We also analyzed the rank abundance curves of all 13 samples (Figure 1D). Unlike what the Shannon index indicated, it was found that both the richness and evenness of the control group were higher than those of the mild DR group (*p* < 0.05).

### Functional Annotation Analysis

**FIGURE 2:**
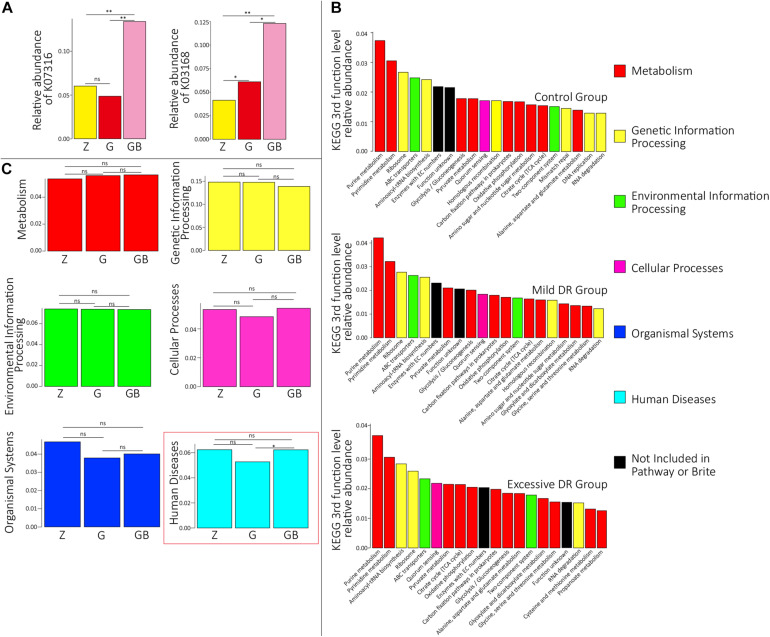
Protein functional annotation analysis. **(A)** KOs with a significant difference in the relative abundance between groups. *Z* for the control group, G for the mild DR group, GB for the excessive DR group. ns: non-significant, *0.01 < *p* < 0.05, **p < 0.01. **(B)** The top 20 KEGG third-level pathways with the highest average relative abundance of each group, different colors in the legend indicate different KEGG first-function-level pathways. **(C)** The difference of relative abundance of each of six KEGG first-function-level pathways among groups. *Z* for the control group, G for the mild DR group, GB for the excessive DR group. ns: non-significant, *p* > 0.05; *0.01 < *p* < 0.05.

The predicted relative abundance of all KOs of each sample is shown in Supplementary Table 2. We visualized the top 20 KOs with the highest average abundance of each group using the R software. In the control group, K07316 (mod, adenine-specific DNA methyltransferase) was the most dominant KO followed by K03168 (top A, DNA topoisomerase I); in the mild DR group, the abundance of K03168 increased and became the most abundant, whereas the abundance K07316 decreased; in the excessive DR group, the abundance of K07316 and K03168 both significantly increased; K07316 became the most abundant KO again and followed by K03168 (Supplementary Figure 2). Then we compared the average relative abundance of the same KOs among different groups. The relative abundance of K07316 and K03168 significantly changed (*p* < 0.05), whereas the other KOs (within the top 20) did not significantly change (*p* > 0.05) (Figure 2A). The detailed data of the predicted relative abundance of all KEGG third-level pathways of each sample are shown in Supplementary Table 3. We also visualized the top 20 KEGG third-level pathways with the highest average abundance of each group using an R software (Figure 2B), and it is difficult to sum up the changing patterns induced by DR among the three groups at KEGG third-level pathways. Then KEGG enrichment analysis was also performed to analyze the differences of KEGG second-level pathways among the groups. We obtained six enriched third-level pathways between the control group and the mild DR group, eight enriched third-level pathways between the mild DR group and the excessive DR group, and 11 enriched third-level pathways between the control group and the excessive DR group (*p* < 0.05) (Table 1). At last, we also visualized the average abundance of 6 KEGG first-level pathways of each group (Figure 2C). Interestingly, the average abundance of the disease pathway of the excessive DR group was significantly higher than the mild DR group (*p* < 0.05), but other pathways did not significantly change (*p* > 0.05).

**TABLE 1:** The results of KEGG enrichment analysis.

Group	Pathway	Pathway name	KEGG level 1	KEGG level 2
Control group vs. mild DR group	ko99984	Nucleotide	Not included in pathway or BRITE	Unclassified: metabolism
	ko00965	Betalain biosynthesis	Metabolism	Biosynthesis of other secondary metabolites
	ko04614	Renin-angiotensin system	Organismal Systems	Endocrine system
	ko00240	Pyrimidine metabolism	Metabolism	Nucleotide metabolism
	ko04622	RIG-I–like receptor signaling pathway	Organismal Systems	Immune system
	ko00910	Nitrogen metabolism	Metabolism	Energy metabolism
Mild DR group vs. excessive DR group	ko00010	Glycolysis/gluconeogenesis	Metabolism	Carbohydrate metabolism
	ko00750	Vitamin B6 metabolism	Metabolism	Metabolism of cofactors and vitamins
	ko00520	Amino sugar and nucleotide sugar metabolism	Metabolism	Carbohydrate metabolism
	ko00350	Tyrosine metabolism	Metabolism	Amino acid metabolism
	ko00340	Histidine metabolism	Metabolism	Amino acid metabolism
	ko99997	Function	Not included in pathway or BRITE	Poorly characterized
	ko00780	Biotin metabolism	Metabolism	Metabolism of cofactors and vitamins
	ko99976	Replication	Not included in pathway or BRITE	Unclassified: gene□c information processing
Control group vs. excessive DR group	ko00760	Nicotinate and nicotinamide metabolism	Metabolism	Metabolism of cofactors and vitamins
	ko00590	Arachidonic acid metabolism	Metabolism	Lipid metabolism
	ko04918	Thyroid hormone synthesis	Organismal Systems	Endocrine system
	ko05133	Pertussis	Diseases	Infectious diseases: bacterial
	ko00523	Polyketide sugar unit biosynthesis	Metabolism	Metabolism of terpenoids and polyketides
	ko00520	Amino sugar and nucleotide sugar metabolism	Metabolism	Carbohydrate metabolism
	ko00521	Streptomycin biosynthesis	Metabolism	Biosynthesis of other secondary metabolites
	ko00051	Fructose and mannose metabolism	Metabolism	Carbohydrate metabolism
	ko00480	Glutathione metabolism	Metabolism	Metabolism of other amino acids
	ko00720	Carbon fixation pathways in prokaryotes	Metabolism	Energy metabolism
	ko00020	Citrate cycle (TCA cycle)	Metabolism	Carbohydrate metabolism

*DR, dietary restriction; KO, KEGG Orthology.*

### Species Composition Annotation Analysis

**FIGURE 3:**
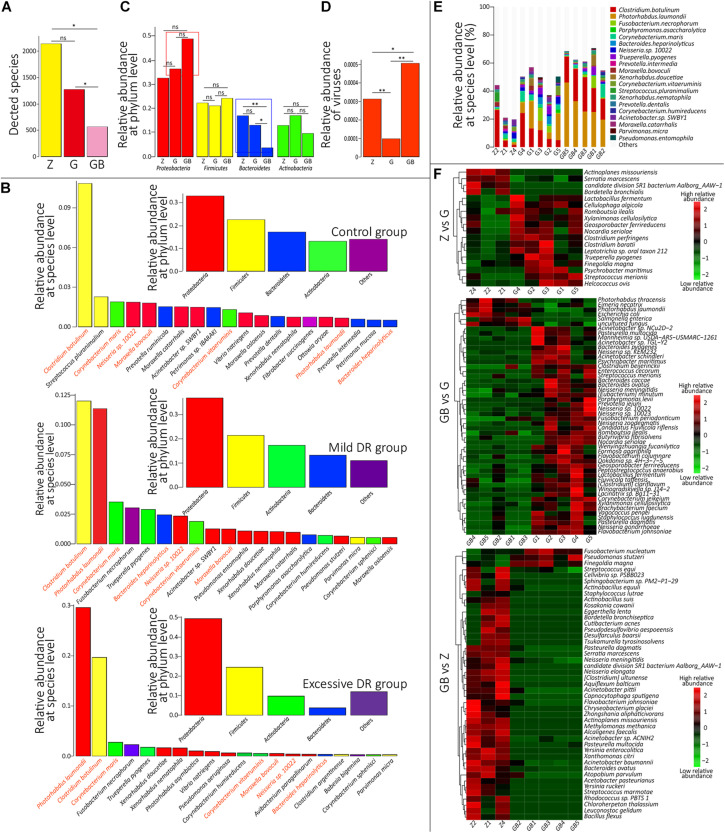
Species annotation analysis. **(A)** The average detected species number of each group. *Z* for the control group, G for the mild DR group, GB for the excessive DR group. ns: non-significant, *p* > 0.05; *0.01 < *p* < 0.05. **(B)** Inner: Top four dominant phyla with the highest average relative abundance in each group. Outer: The top 20 dominant species with the highest average relative abundance in each group. Species with the same color belong to the phyla with the corresponding color. Species name marked by orange means “core species.” **(C)** The difference of average relative abundance of each of four dominant phyla in each group. *Z* for the control group, G for the mild DR group, GB for the excessive DR group. ns: non-significant, *0.01 < *p* < 0.05; **p < 0.01. **(D)** The difference of average relative abundance of the detected virus number in each group. *Z* for the control group, G for the mild DR group, GB for the excessive DR group. ns: non-significant, *0.01 < *p* < 0.05; **p < 0.01. **(E)** The top 20 dominant species with the highest average relative abundance in each sample and their percentages, *Z* for the control group, G for the mild DR group, GB for the excessive DR group. **(F)** Heat map shows the top 50 species with the highest significance among groups. *Z* for the control group, G for the mild DR group, GB for the excessive DR group. Red indicates that the species has higher relative abundance, whereas green indicates that the species has lower relative abundance. The value is the result of the *Z* scoring of species relative abundance; the bigger value of color, the higher relative abundance.

**FIGURE 4:**
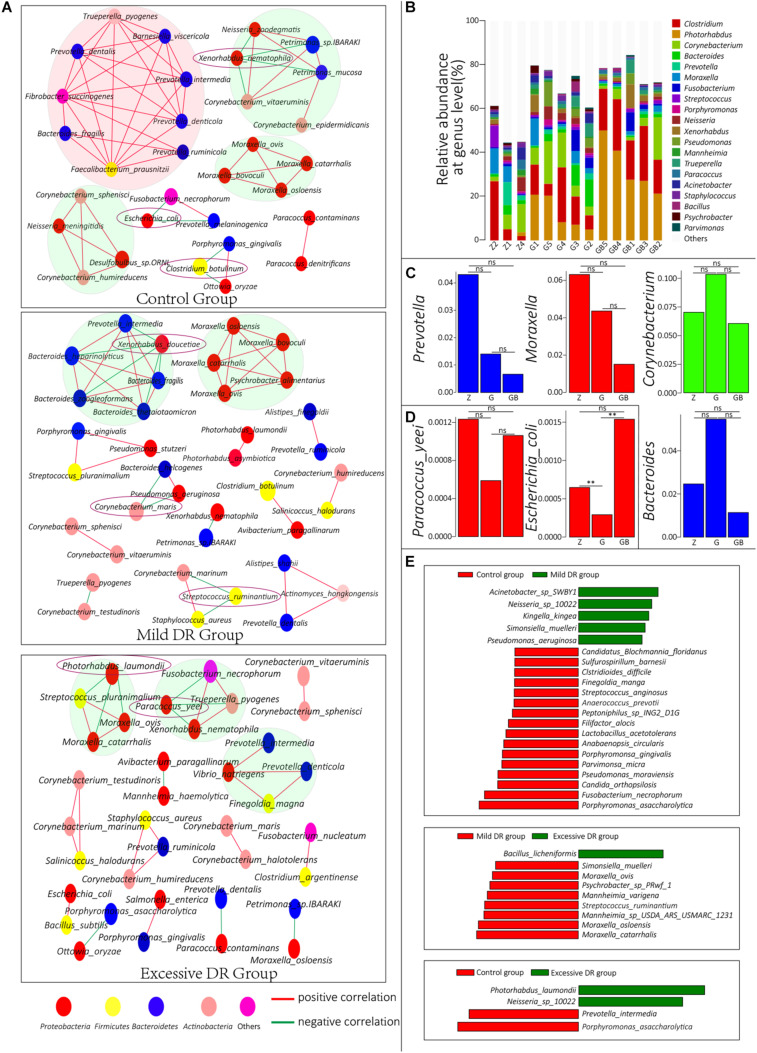
**(A)** Co-correlation network of the top 50 species with the highest average relative abundance in each group. One circle indicates one species, and the species with the same color belong to a phylum with the corresponding color. **(B)** The top 20 dominant phyla with the highest average relative abundance in each sample and their percentages, *Z* for the control group, G for the mild DR group, GB for the excessive DR group. **(C,D)** The difference of average relative abundance of some key species and genus in each group, *Z* for the control group, G for the mild DR group, GB for the excessive DR group. ns: non-significant; *0.01 < *p* < 0.05; **p < 0.01. **(E)** Linear discriminant analysis (LDA) effect size (LEfSe) analysis between groups. The red square indicates that the relative abundance increased, and the green square indicates that the relative abundance decreased. The length of the square indicates the magnitude of significance.

At the species level, 4,271 microbial taxa were detected in all three groups, and the detailed relative abundance data at the species level in each sample are shown in Supplementary Table 4. We counted the average number of detected species in each group and found that the number of detected species was significantly lower in the excessive DR group than in the other two groups (*p* < 0.05) and the species number of the mild DR group also significantly lower than in the control group (*p* < 0.05) (Figure 3A). At the phylum level, the top four phyla with the highest average relative abundance in each group were identified (Figure 3B), and their variations among the groups were analyzed (Figure 3C). In the control group, *Proteobacteria* was the most dominant phylum, followed by *Firmicutes*, *Bacteroidetes*, and *Actinobacteria*; in the mild DR group and excessive DR group, *Proteobacteria* was also the most dominant phylum and followed by *Firmicutes*, *Actinobacteria*, and *Bacteroidetes*. It is found that the abundance of *Bacteroidetes* varied most significantly (*p* < 0.05), the abundance of *Proteobacteria* showed a trend of variation (*p* < 0.07) between the control group and excessive DR group, and the abundance of *Firmicutes* and *Actinobacteria* did not differ significantly among groups (*p* > 0.05). The detailed relative abundance data of each sample are shown in Supplementary Table 5. To analyze the species composition more intuitively, we visualized the top 20 species with the highest relative abundance of each group (Figures 3B,E). *Clostridium botulinum* was the most dominant species in the control group and mild DR group, whereas *Photorhabdus laumondii* was the most dominant species in the excessive DR group (Figures 3B,E). The abundance of other species was distinctly decreased in the mild DR group (G: 45% vs. Z: 69%) and excessive DR group (GB: 25% vs. Z: 69%) (Figure 3E). And to analyze the difference in species composition among groups, we compared the average relative abundance of the same species among different groups (a total of 34 different kinds of top 20 abundant species in all three groups) (Supplementary Figure 3A). We identified five species, which significantly changed among three groups: *P. laumondii*, *Avibacterium paragallinarum*, *Babesia bigemina*, *Pseudomonas stutzeri*, and *Neisseria* sp. 10022 (Supplementary Figure 3A); 7 of these 34 species were at present in all three groups of top 20 abundant species: *C. botulinum*, *P. laumondii*, *Corynebacterium maris*, *Bacteroides heparinolyticus*, *Neisseria* sp. 10022, *Corynebacterium vitaeruminis*, and *Moraxella bovoculi* (Figure 4B and Supplementary Figure 3B), which might be “the core bacteria” for the yak nasopharynx microbial community. We also detected the species number of viruses in each group and their average relative abundance (Supplementary Table 6 and Figure 3D). The average species number of viruses detected in the mild DR group and excessive DR group was much lower than that in the control group (8, 9, and 23, respectively) (Supplementary Table 6). However, the average relative abundance of detected virus in the mild DR group was extremely lower than that in the control group (*p* < 0.01), and the average relative abundance of the excessive DR group was extremely higher than that in the mild DR group (*p* < 0.01), whereas the average relative abundance in the excessive DR group was significantly higher than that in the control group (*p* < 0.05) (Supplementary Table 6 and Figure 3D). Meanwhile, to analyze the abundance variation among groups more generally, we also performed a cluster analysis for the top 50 species with the highest significance (*p* < 0.05) of variation and drew a heat map to visualize the results (Figure 3F and Supplementary Table 7). Against the control group, mild DR significantly altered 18 species (4 were decreased, 14 were increased); against the control group, excessive DR significantly decreased 47 of the 50 species and significantly increased the other 3 of them, and against the mild DR group, excessive DR significantly decreased 44 of the 50 species and increased the other 6 of them. It is worth noting that there were two alteration patterns: one is that the species alteration induced by 7 days of DR quickly recovered after 2 days of BHBA intravenous injection treatment (P1 and P2 in Supplementary Figure 3A); the other one is that the species alteration induced by 7 days of DR was further enhanced after 2 days of BHBA intravenous injection treatment (P3 and P4 in Supplementary Figure 3A).

### Microbial Interactions Prediction Analysis

To understand the interrelationships among these microorganisms in each group, we constructed co-connection networks of the top 50 species with the most dominant abundance in each group, and the results showed that the species interconnection within the control group was tighter than the mild DR group or excessive DR group (Figure 4A). Interestingly, most of these species had a positive correlation with each other (red line), whereas only a few of these species had a negative correlation with other species (green line). In the control group, there were four dominant teams (marked by big circles), which mainly consisted of genus *Prevotella*, *Moraxella*, and *Corynebacterium*. Within these 50 species, *Xenorhabdus nematophila*, *Escherichia coli*, and *C. botulinum* had a negative correlation with other species and could be regarded as “key species.” In the mild DR group, similarly, there were two dominant teams, which were mainly composed of genus *Bacteroides* and *Moraxella*. *Xenorhabdus doucetiae*, *C. maris*, and *Streptococcus ruminantium* were the “key species.” In the excessive DR group, there were three dominant teams (no major genera); *P. laumondii* and *Paracoccus yeei* were the “key species.” We also counted and visualized the top 20 genera with the highest relative abundance of each sample. It was found that *Clostridium* was the most dominant genus in the control group, whereas *Photorhabdus* was the most dominant genus in the mild DR group and excessive DR group (Figure 4B). Then, we compared the average relative abundance of the same genus (Figure 4C) and the “key species” (Supplementary Figure 3A and Figure 4D), which was mentioned previously. It was found that these major genera did not significantly change (*p* > 0.05) among groups, but two kinds of “key species” had been significantly changed (*p* < 0.05) among groups: *P. laumondii* and *E. coli*. The detailed data are shown in Supplementary Table 8 (Sheet 1). And finally, to find a biomarker species of each group, we performed a LEfSe analysis between groups and identified the five species with the most significant relative abundance difference as the biomarker species: *Acinetobacter* sp. SWBY1, *Porphyromonas asaccharolytica*, *Bacillus licheniformis*, *Moraxella catarrhalis*, and *P. laumondii* (Figure 4E).

## Discussion

In the present study, the influence of starvation on the nasopharyngeal microbiome was explored, and its potential mechanism was discussed. Metagenomic sequencing identified the four most dominant phyla: *Proteobacteria*, *Firmicutes*, *Bacteroidetes*, and *Actinobacteria*; five most dominant genera: *Clostridium*, *Bacteroides*, *Prevotella*, *Moraxella*, and *Streptococcus*; and the most dominant species: *C. botulinum*. From a protein perspective, starvation mainly affected K07316 and K03168, which were once discussed by Desirazu et al. (Rao et al., 2014) and Giovanni et al. (Capranico et al., 2017), respectively. From a species perspective, starvation mainly affected *Proteobacteria* and *Bacteroidetes* at the phyla level. Whereas mild DR affected some KOs and species but had no significant influence on the nasopharyngeal microbiota community, excessive DR significantly decreased the diversity of the community by affecting oral microorganisms, and disturbed their composition and structure, implying a higher risk of respiratory tract diseases.

### Intravenous Injection of BHBA Simulates Severe DR

The 9 days of DR group (G) and BHBA intravenous injection group (GB) represented short-term mild DR and long-time excessive DR, respectively. Generally, animals would go through three stages as the degree of starvation increases. First, when an animal cannot take sufficient food in, it will use stored glycogen or synthesize glucose by gluconeogenesis to maintain a certain concentration of blood glucose that supplies the necessary energy for some essential physiological functions. Yu et al. (2016) and Zou et al. (2019) found that in the first 9 days of starvation, the blood glucose concentration in the yak would significantly decrease immediately and then remains stable. Second, when the stored glycogen runs out, the animal body will break fat down to provide energy. BHBA, the main product of fat catabolism, is preferentially utilized by the brain and nervous system (Prince et al., 2013). Zou et al.s’ (2020) had confirmed that BHBA intake after 7 days of starvation significantly increased the blood BHBA concentration in yaks. For instance, in the perinatal period, cows often experience ketoacidosis due to large amounts of fat catabolism induced by severe nutrient deficiency (Suthar et al., 2013). The main characteristics of ketoacidosis are the high concentration of BHBA and low concentration of blood glucose (Frise et al., 2013; Jezek et al., 2017; McIntyre et al., 2019). Third, when the fat runs out, proteins in the animal tissues will begin to be degraded, which can lead to serious consequences, even death. Hence, BHBA intravenous injection treatment was performed using the same procures as Zou et al.s’ (2020) experiment of Zou and was similar to that induced by long-term starvation or lactation.

### Mild Starvation Slightly Affects the Microbial Community of the Nasopharynx Probably by Altering the Oral Microbiota and Mucosal Mucins

Because we controlled environmental factors, the mucosal immune system of the host and available energy resources are the two major internal factors that determine the homeostasis of the nasopharyngeal microbial community (Brugman and Nieuwenhuis, 2010; David et al., 2014). The enrichment analysis results showed that the expression level of the immune system, nucleotide metabolism, and secondary metabolites pathways in the mild DR group were significantly down-regulated when compared with the control group, indicating that mild DR affected the proliferation and metabolism of the community. And the changes in energy metabolism also indicated that their energy source had changed (Table 1). Besides there was a more complex network consisting of *Prevotella* in the control group (Figure 4A). *Prevotella* mainly exists in the digestive system and absorbs nutrients by breaking down cellulose (Suthar et al., 2013; Kovatcheva-Datchary et al., 2015). But this network faded away gradually as the DR level increased (Figure 4A). Although it is insignificant, the relative abundance of genus *Prevotella* was decreased in both the mild DR group and excessive DR group when compared to the control group (Figure 4C).

Because of the topographical continuity between the oral cavity and nasopharynx, the microorganisms of the oral cavity can spread to the nasopharynx (Charlson et al., 2011). Considering the main nutrient source of *Prevotella* is fiber (Kovatcheva-Datchary et al., 2015), which comes from fodder, we speculated that the decrease of *Prevotella* relative abundance in the oral cavity induced by DR led to its decrease in the nasopharynx. When yaks cannot take in fodder or pasture, *Prevotella* in the oral cavity cannot obtain fiber, leading to the abundance of oral *Prevotella* decrease, and so does the nasopharynx *Prevotella*. Also, *P. asaccharolytica*, the common oral cavity bacteria, was the common biomarker (Figure 4E) for the control group against both the mild DR group and excessive DR group, also indicating that oral microorganisms had a tighter connection with nasopharyngeal bacteria.

It was also shown that the relative abundance of some other species increased in the mild DR group, such as *Clostridium* and *Photorhabdus*, which are resident bacteria in cattle respiratory tracts (Holman et al., 2015; Lima et al., 2016). In the mild DR group, the blood glucose concentration was very low, indicating a decrease in mucosal mucin secretion and insufficient mucosal immune function to manage the microbiota (Marcos et al., 2003). Frenkel and Ribbeck (2017) found that salivary mucins affected the bacterial viability by promoting a less competitive growth mode, and Flynn et al. (2016) confirmed that *Pseudomonas aeruginosa* could degrade mucins into nutrients, and mucins are essential for some pathogens. Therefore, we thought that the decrease of normal fiber-degrading oropharynx bacteria would empty the ecological niche in the nasopharynx. The empty ecological niche could provide suitable proliferation resources for those mucin-degrading bacteria and pathogens, resulting in those already decreased mucins being further consumed. Finally, the homeostasis of the nasopharyngeal microbial community would be destroyed.

The co-connection network results also showed that DR mainly affected the team in the pink big circle. *Faecalibacterium prausnitzii*, which is a widely accepted probiotic for humans (Ferreira-Halder et al., 2017; Lopez-Siles et al., 2017), and the five other commensal oral fiber-degrading bacteria: *Prevotella dentalis*, *Fibrobacter succinogenes*, *Prevotella ruminicola*, *Prevotella denticola*, and *Prevotella intermedia* (Kobayashi et al., 2008), consisting of the main team in the control group. And the three biomarkers species, *Acinetobacter* sp. SWBY1, *P. asaccharolytica*, and *B. licheniformis*, also were common oral bacteria.

Taking all these evidence into account, two change patterns were concluded. First, oropharynx-derived microorganisms and their collaborators have decreased. Second, microorganisms competing for the ecological niche with oropharyngeal microbiota and microorganisms inhibited by mucins have increased.

### Excessive DR Significantly Altered the Homeostasis of the Nasopharyngeal Microbial Community Because of the Presence of BHBA

BHBA treatment significantly decreased the diversity and affected the homeostasis of the nasopharyngeal microbial community. Compared with the control group, the pathways of energy metabolism, secondary metabolites biosynthesis, and carbon fixation in the excessive DR group were changed in a wider range than those in the mild DR group (Table 1), indicating that excessive DR had a stronger influence on the community function. By combining the data of both the mild DR group and excessive DR group, we concluded four alteration patterns: after 2 days of BHBA intravenous injection treatment, the increase and decrease that induced by 7 days of DR were recovered or were further enhanced (Supplementary Figure 3A). Yaks in the mild DR group and excessive DR group were treated with the same operations, except that those in the excessive DR group were treated with intravenous infusion of BHBA instead of normal saline. Therefore, we speculated that the presence of BHBA was the reason for the diversity decreasing and homeostasis alteration in the excessive DR group. Like subclinical ketosis, BHBA treatment increased not only the glucose concentration but also the BHBA concentration in blood (Andersson, 1988; Sturm et al., 2020) and resulted in increased ketone bodies in the exhalant gas (Dobbelaar et al., 1996), which could be used as nutrients by some bacteria. Schulz et al. (2015) found that cows with subclinical ketosis showed an enhanced immune response when compared with metabolically healthy individuals. Zou et al.s’ (2020) study also confirmed that BHBA treatment recovered the concentration of blood sugar. This enhanced immune response and recovered blood glucose indicated that BHBA treatment recovered the immune system and the secretion of mucosal mucins, inhibiting those bacteria without mucin resistance. Because the lack of fodder and pasture doesn’t recover, the decrease of normal oropharynx fiber-degrading bacteria continuous, and then this empty ecological niche would be occupied by those bacteria which could utilize mucins or ketone bodies as energy resources. Ketones and mucins improved the proliferation of some bacteria, while the enhanced immune response inhibited the proliferation of some others, which resulted in these four alteration patterns mentioned previously (Supplementary Figure 3A). Therefore, BHBA, which is a more efficient energy resource, replenished the energy needs of the DR yaks and enhanced the immune system, but did not alter the lack of normal oropharynx bacteria, finally resulting in the extremely complex alterations and these significant influences.

### Excessive DR Increased the Risk of Respiratory Diseases

From a protein perspective, excessive DR down-regulated the biosynthesis metabolism of streptomycin, which has a powerful antibacterial effect (Schatz et al., 2005). Otherwise, excessive DR up-regulated bacterial infection-related pathways such as pertussis (de Gouw et al., 2011), which is a common bovine respiratory disease, and the up-regulated KEGG first-level pathway in the diseases (KEGG BRITE: 08402) (Figure 2C). All these results indicated that BHBA treatment increased the risk of diseases including respiratory tract diseases. The same conclusion can be drawn from the view of observed species alteration. Excessive DR treatment significantly decreased the microbial diversity, which means the risks of respiratory flora disorders and respiratory diseases were increased (Koppen et al., 2015; Dickson et al., 2016; Man et al., 2017; Zeineldin et al., 2019). BHBA treatment also increased the relative abundance of *Proteobacteria* (Figure 3C), which is considered as a common factor of inflammation and lung diseases (Rizzatti et al., 2017). Correlation network analysis (Figure 4A) results showed that excessive DR destroyed the microbial community interrelationship in the control group, and this disorder was thought to contribute to respiratory diseases (Koppen et al., 2015; Zeineldin et al., 2019). Our results showed that BHBA treatment increased the relative abundance of *Pseudomonas Acinetobacter*, *Bacillus*, *Bacteroides*, *Clostridium*, and *Enterococcus*, which are common bovine respiratory pathogens (Klima et al., 2019). Moreover, BHBA treatment significantly increased the relative abundance of viruses (including bacteriophage) and decreased their kind number (Supplementary Table 6), indicating that BHBA treatment increased the risk of respiratory diseases. All these evidences indicated that BHBA treatment could increase the risk of respiratory diseases.

Furthermore, those “key species” negatively correlated with most of the other species (within the top 50); it was speculated that these species might play an important role in maintaining the homeostasis of the nasopharyngeal microbial community. *F. prausnitzii* and other *Prevotella* bacteria, which formed the biggest connection network in the control group, were considered to be probiotics by some researchers (Ley, 2016; Lopez-Siles et al., 2017). They and those biomarkers of the control group might be useful in the prevention and treatment of bovine respiratory diseases. Nevertheless, further evidence is still needed.

## Conclusion

In summary, although we simulated excessive DR by using BHBA intravenous injection treatment instead of really testing excessive starvation, the present study was sufficient to confirm that starvation would affect the composition, function, and diversity of the yak nasopharyngeal microbial community. Starvation mainly affected *Bacteroidetes* and *Proteobacteria* at the phylum level, whereas *P. laumondii*, *A, paragallinarum*, *B. bigemina*, *P. stutzeri*, and *Neisseria* sp. 10022 at the species level. The influence of mild starvation was insignificant. Excessive starvation affected the oral microorganisms and mucosal mucins, and significantly disturbed the nasopharynx microbiome, and increased the risk of respiratory diseases. These results could enrich our knowledge of the respiratory tract microenvironment and provide us with new strategies for respiratory disease prevention and treatment. However, because of the lack of longitudinally following these yaks, physiological data, and the limitation of sample size, further experiments are still required.

## Data Availability Statement

The datasets presented in this study can be found in online repositories. The names of the repository and accession number can be found below: NCBI SRA database; accession number is PRJNA681085.

## Ethics Statement

The animal study was reviewed and approved by Institutional Animal Care and Use Committee of Sichuan Agricultural University.

## Author Contributions

JQ, ZW, and ZZ conceived and designed the experiments. JQ, HZ, ZZ, and YC performed the experiments. JQ, YC, ZZ, and DC analyzed the data and wrote the manuscript. All authors critically reviewed the manuscript.

## Conflict of Interest

The authors declare that the research was conducted in the absence of any commercial or financial relationships that could be construed as a potential conflict of interest.

## Footnotes

1 https://www.ncbi.nlm.nih.gov/bioproject/PRJNA681085

## Supplementary Material

Supplementary Figure 1 **(A)** The PCA (principal component analysis) of KEGG Orthology (KO). Fifteen samples can be roughly clustered into two groups (pink and reseda) like species PCA. **(B)** The flowchart of procedures in the experiment before sampling.

Supplementary Figure 2 The average relative abundance of top 20 KOs with the highest relative abundance in each group.

Supplementary Figure 3 **(A)** The difference of average relative abundance of 34 top 20 species, which occurred in all three groups. Species with the same color belong to the phyla with the corresponding color like Figure 4. Z for the control group, G for the mild DR group, GB for the excessive DR group. ns: non-significant, *p* > 0.05; *0.01 < *p* < 0.05. Marking by the red square means the difference was significant. The yellow line divided these 34 species into four groups according to their changing rule. P1: pattern 1, BHBA treatment enhanced the increase of relative abundance; P2: pattern 2, BHBA treatment enhanced the decrease of relative abundance; P3: pattern 3, BHBA recovered the decrease of relative abundance; P4, pattern 4, BHBA treatment recovered the increase of relative abundance. **(B)** Venn diagram analysis of 34 top 20 species of three groups. Red words indicate that the relative abundance of this species increased; purple words indicate that the relative abundance of this species decreased. The overlapping parts of the circles represent the species that are shared in corresponding groups.

Supplementary Table 1 **(A)** Statistical table of reads data. N (%): the ratio of fuzzy bases to total bases; GC (%): the ratio of G and C bases to total bases; Q20 (%): the proportion of bases with accuracy above 99% to total bases; Q30 (%): the proportion of bases with accuracy above 99.9% to total bases; HQ reads (%): the percentage of high-quality sequences in the raw sequences; HQ data (%): the percentage of bases in high quality-sequences to the bases in raw sequences. **(B)** The detailed Simpson index, Chao1 index, ACE index, and Shannon index of each sample and their average means of each group.

Supplementary Table 2 Detailed relative abundance of KOs of each sample.

Supplementary Table 3 Detailed relative abundance of all KEGG third-level pathways of each sample.

Supplementary Table 4 Detailed relative abundance of every species of each sample.

Supplementary Table 5 Detailed relative abundance data at the phyla level of each sample.

Supplementary Table 6 Detailed relative abundance data of detected viruses of each group.

Supplementary Table 7 Detailed relative abundance data of top 50 species significantly changed species.

Supplementary Table 8 **(A)** Detailed relative abundance data at the genus level of each sample. **(B)** Detailed statistical information of contigs/scaffolds. Length of N20/50/90: arranges all the assembled contigs/scaffolds sequences in order of length from long to short and then added from long to short. When the addition length reaches 20%/50%/90% of the total length of contigs/scaffolds sequences, the corresponding length of the last series will be the length of N20/50/90.
