# Supplementary figures and images for: Metagenomics Reveals That Intravenous Injection of Beta-Hydroxybutyric Acid (BHBA) Disturbs the Nasopharynx Microflora and Increases the Risk of Respiratory Diseases
Source: Front Microbiol. 2021 Feb 5;11:630280. doi: 10.3389/fmicb.2020.630280 (PMC7892611; doi:10.3389/fmicb.2020.630280)

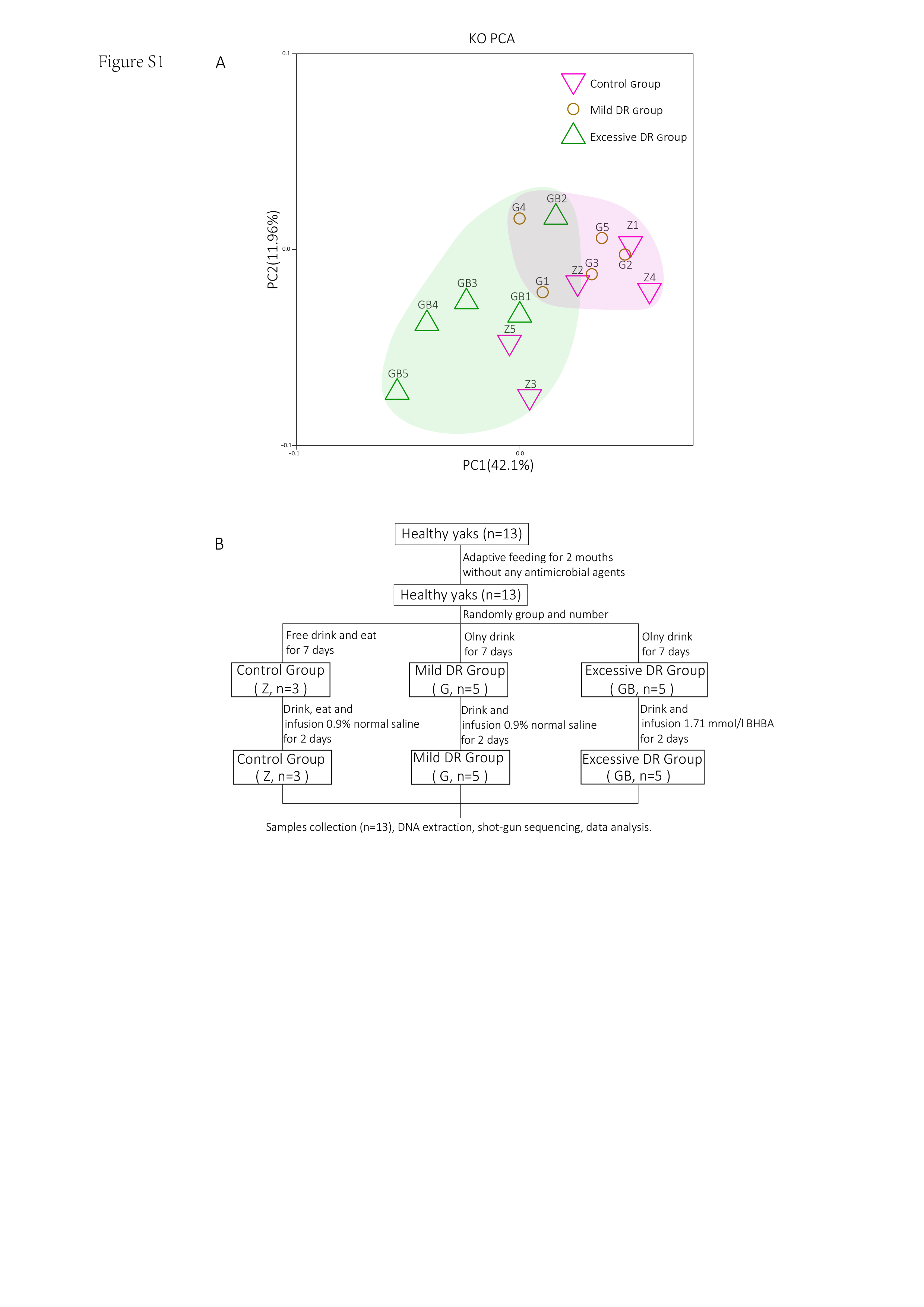

Supplement: Supplementary Figure 1 — (A) The PCA (principal component analysis) of KEGG Orthology (KO). Fifteen samples can be roughly clustered into two groups (pink and reseda) like species PCA. (B) The flowchart of procedures in the experiment before sampling. [file Image_1.TIF]

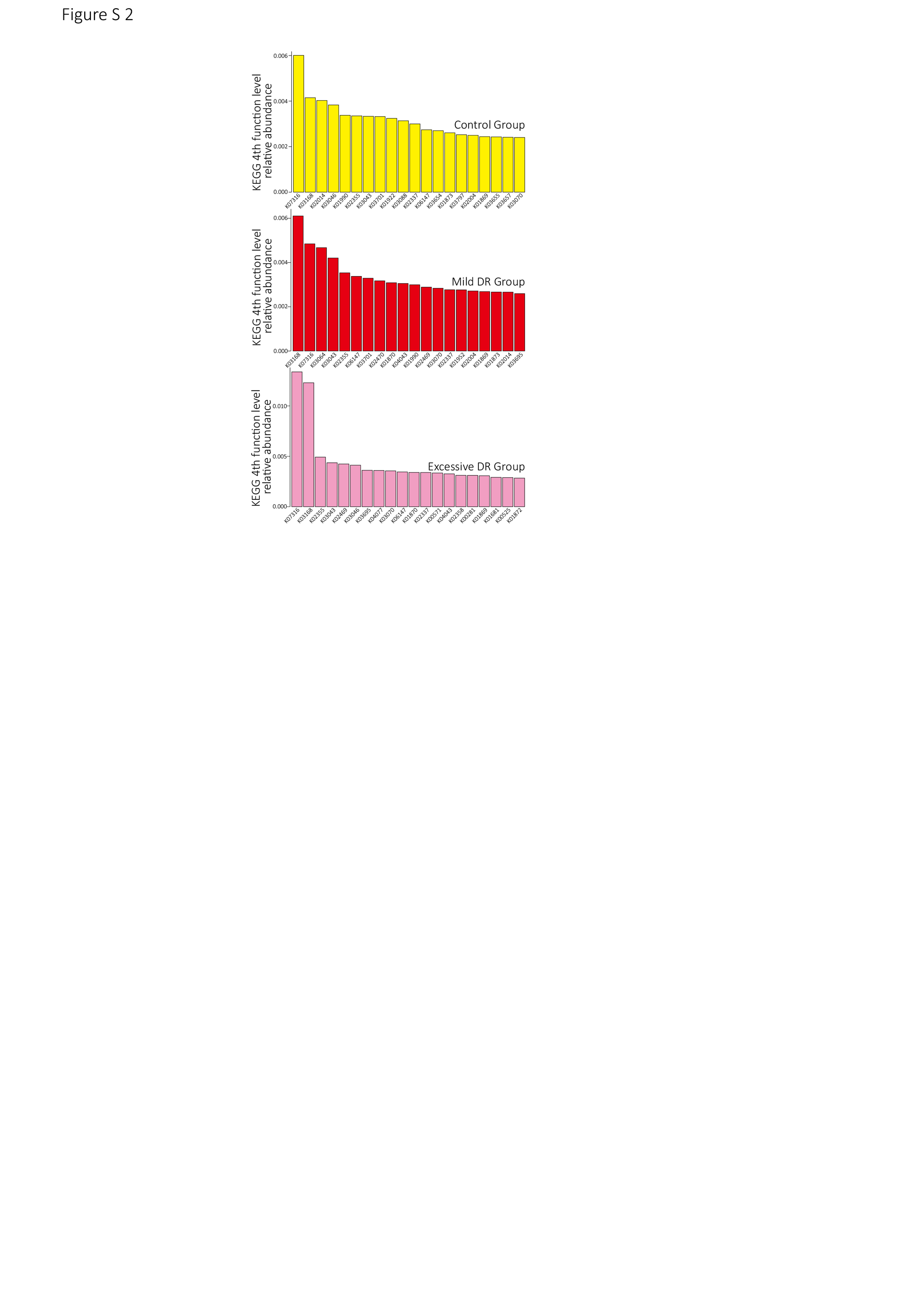

Supplement: Supplementary Figure 2 — The average relative abundance of top 20 KOs with the highest relative abundance in each group. [file Image_2.TIF]

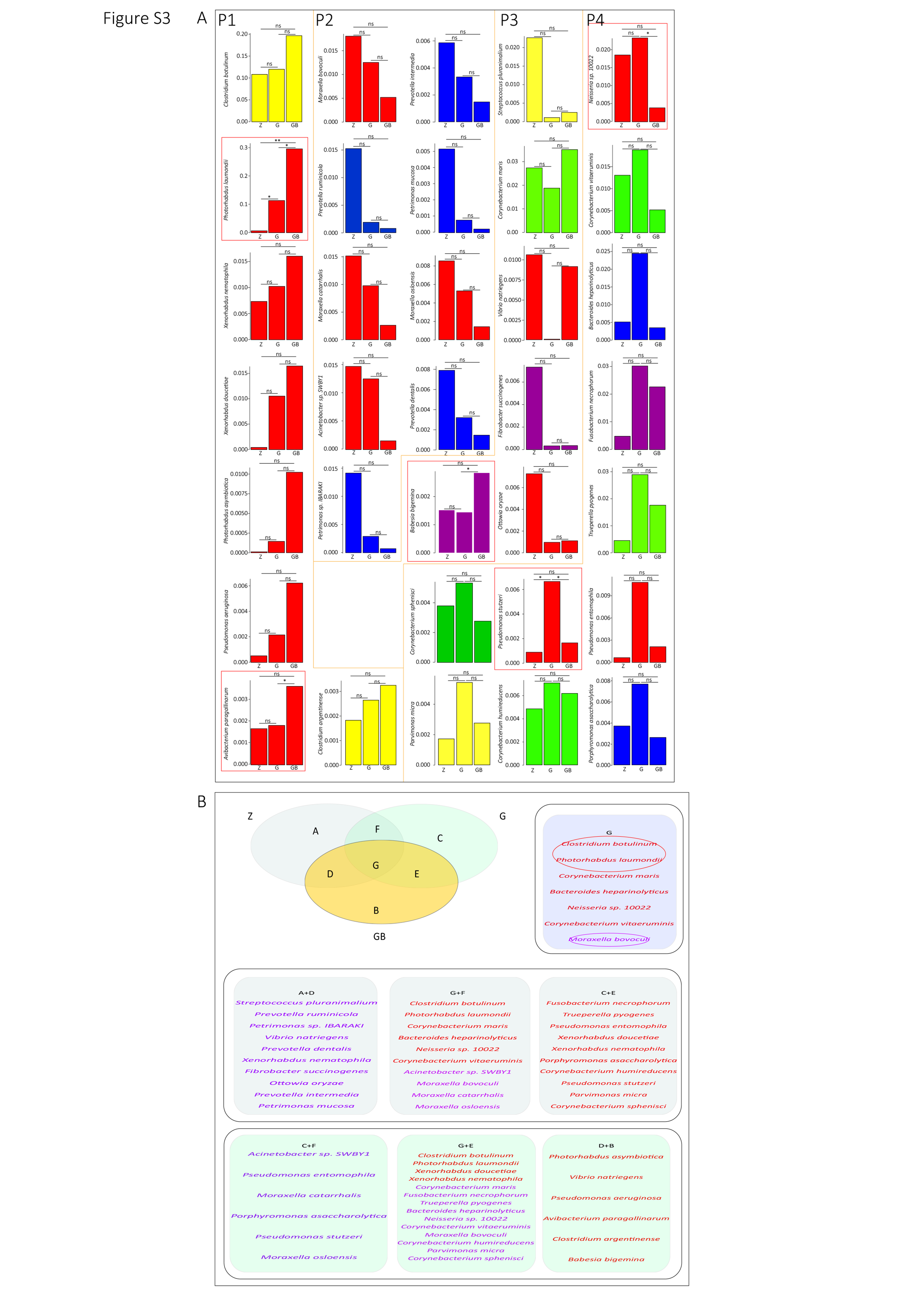

Supplement: Supplementary Figure 3 — (A) The difference of average relative abundance of 34 top 20 species, which occurred in all three groups. Species with the same color belong to the phyla with the corresponding color like Figure 4. Z for the control group, G for the mild DR group, GB for the excessive DR group. ns: non-significant, p > 0.05; *0.01 < p < 0.05. Marking by the red square means the difference was significant. The yellow line divided these 34 species into four groups according to their changing rule. P1: pattern 1, BHBA treatment enhanced the increase of relative abundance; P2: pattern 2, BHBA treatment enhanced the decrease of relative abundance; P3: pattern 3, BHBA recovered the decrease of relative abundance; P4, pattern 4, BHBA treatment recovered the increase of relative abundance. (B) Venn diagram analysis of 34 top 20 species of three groups. Red words indicate that the relative abundance of this species increased; purple words indicate that the relative abundance of this species decreased. The overlapping parts of the circles represent the species that are shared in corresponding groups. [file Image_3.TIF]
