# Supplementary material for: Metagenomics Reveals That Intravenous Injection of Beta-Hydroxybutyric Acid (BHBA) Disturbs the Nasopharynx Microflora and Increases the Risk of Respiratory Diseases
Source: Front Microbiol. 2021 Feb 5;11:630280. doi: 10.3389/fmicb.2020.630280 (PMC7892611; doi:10.3389/fmicb.2020.630280)
Supplement: Supplementary Table 1 — (A) Statistical table of reads data. N (%): the ratio of fuzzy bases to total bases; GC (%): the ratio of G and C bases to total bases; Q20 (%): the proportion of bases with accuracy above 99% to total bases; Q30 (%): the proportion of bases with accuracy above 99.9% to total bases; HQ reads (%): the percentage of high-quality sequences in the raw sequences; HQ data (%): the percentage of bases in high quality-sequences to the bases in raw sequences. (B) The detailed Simpson index, Chao1 index, ACE index, and Shannon index of each sample and their average means of each group. [file Table_1.pdf]

Table S1

A

sample	reads	bases	N(%)	GC(%)	Q20(%)	Q30(%)	HQ reads(%)	HQ data(%)
Z1	58647206	8797080900	0.00050	44.55	96.74	91.66	99.87	98.74
Z2	59243294	8886494100	0.00052	44.10	96.79	91.77	99.87	98.78
Z3	58022568	8703385200	0.00051	44.55	96.71	91.58	99.86	98.75
G1	42558414	6383762100	0.00052	44.36	96.71	91.73	99.85	98.62
G2	57766548	8664982200	0.00051	44.22	96.74	91.67	99.86	98.75
G3	47370166	7105524900	0.00051	44.26	96.74	91.66	99.87	98.75
G4	46240952	6936142800	0.00051	44.09	96.62	91.46	99.86	98.67
G5	58700414	8805062100	0.00051	45.19	96.98	92.08	99.89	98.94
GB1	45485312	6822796800	0.00051	44.54	96.40	91.00	99.85	98.58
GB2	45606992	6841048800	0.00052	44.29	96.41	90.99	99.85	98.60
GB3	49355106	7403265900	0.00051	44.46	96.86	91.99	99.87	98.71
GB4	50242654	7536398100	0.00047	44.31	96.77	89.41	99.86	98.58
GB5	58979374	8846906100	0.00051	45.75	96.29	90.79	99.83	98.54

B

sample	simpson	chao1	ACE	shannon
Z1	0.987680045588	3262.97894737	3138.67067542	8.26444541089
Z2	0.914091972926	1006.32608696	1058.7273298	5.83211012026
Z3	0.985817697315	3138.35714286	3079.16498423	7.98361899825
Mean±SEM	0.96±0.04	2469±1268	2426±1184	7.36±1.33
G1	0.933480701889	1230.0	1451.4216394	5.70485795898
G2	0.980077413035	2724.21212121	2746.64394195	7.56212674836
G3	0.961706614852	1514.7625	1777.1211615	6.41574749199
G4	0.923092078136	1421.84615385	1477.94961595	6.02754909394
G5	0.943820823744	1701.24418605	1847.6186861	5.99264882594
Mean±SEM	0.95±0.02	1718±587	1860±526	6.34±0.73
GB1	0.887234200927	689.675675676	773.998134124	4.49269598904
GB2	0.925009700325	1412.5326087	1655.73733074	5.8204070513
GB3	0.868160481464	851.52	982.099122747	4.71190579809
GB4	0.829719158665	638.115384615	771.384897277	4.0988758725
GB5	0.745042731616	496.018518519	608.478972081	3.40184611007
Mean±SEM	0.85±0.07	818±356	958±412	4.51±0.89
